# Crosstalk between metabolic reprogramming and microbiota: implications for cancer progression and novel therapeutic opportunities

**DOI:** 10.3389/fimmu.2025.1582166

**Published:** 2025-05-20

**Authors:** Xingchen Li, Yidi Jia, Yanqing Li, Hu Hei, Songtao Zhang, Jianwu Qin

**Affiliations:** ^1^ Department of Thyroid Head and Neck Surgery, Affiliated Cancer Hospital of Zhengzhou University, Henan Cancer Hospital, Zhengzhou, Henan, China; ^2^ Public Laboratory, Tianjin Medical University Cancer Institute and Hospital, National Clinical Research Center for Cancer, Key Laboratory of Cancer Prevention and Therapy, Tianjin’s Clinical Research Center for Cancer, Tianjin, China

**Keywords:** anti-tumor therapy, metabolic reprogramming, microbiota, tumor microenvironment, cancer progression

## Abstract

Metabolic reprogramming is a process by which cells adapt to the nutrient microenvironment by regulating energy metabolism. Compared with normal cells, tumor cells tend to undergo metabolic reprogramming, which is one of the hallmarks of concurrent genomic instability, and immune evasion in tumor cells. The microbial community, known as “second genome” of human beings, can cause systemic disease by predisposing cells to tumors, and modulating immune responses to cancer. Metabolic reprogramming and microorganisms can crosstalk with each other in multiple ways to influence various physiological and pathological responses in cancer progression. The products of increased synthesis by tumor cells can reach the intestinal tract via the circulation and act on the microorganisms, promoting mucosal inflammation, causing systemic disorders, and may also regulate the immune response to cancer. In addition, the metabolites of the microorganisms can in turn be transported to the tumor microenvironment (TME) through the systemic circulation and participate in the process of tumor metabolic reprogramming. Different molecular mechanisms related to metabolic reprogramming and microbiota imbalance control the outcome of tumor or anti-tumor responses, depending on the type of cancer, stage of the disease and the TME. In this review, we focus on the fundamental role of metabolic reprogramming in the interaction between microorganisms and cancers and explore the molecular mechanisms by which metabolic reprogramming modulates this complex biological process. This comment aims to provide valuable resources for clinicians and researchers and promote further research in the field.

## Background

Metabolism encompasses complex biochemical networks that convert nutrients into metabolites (1), enabling cells to generate energy, synthesize macromolecules, and maintain cellular functions (2). The most prominent biological characteristic of tumor cells is their uncontrolled proliferation. To meet the biosynthetic demands of rapid growth, cancer cells exhibit distinct metabolic patterns compared with normal cells. Under the influence of factors such as the harsh tumor microenvironment (TME), the metabolic characteristics of cancer cells undergo adaptive changes, which is called metabolic reprogramming (3), and it is one of a hallmark of malignancy (4). This phenomenon was first described by Otto Warburg, who observed that cancer cells preferentially utilize aerobic glycolysis even under oxygen-rich conditions (the Warburg effect) (5). Subsequent research has revealed that metabolic reprogramming extends far beyond the Warburg effect, involving diverse pathways including fatty acid synthesis, glutamate metabolism, and other complex biochemical processes (2, 6). Importantly, these metabolic alterations are dynamic, evolving throughout cancer progression (4). Furthermore, emerging evidence indicates that cancer cells are able to reshape the TME and suppress anti-tumor immune response by depleting essential nutrients (7, 8).

Despite years of research and significant advances in cancer prevention, diagnosis, and treatment, cancer remains a major health burden worldwide (9). As a complex disease, cancer development involves dynamic interactions between tumor cells and the TME (10), with emerging evidence highlighting the critical role of microorganisms (11). It has been found that multiply types of cancers are associated with microbiota, including in breast cancer, lung cancer, gastric cancer, ovarian cancer, and et. al (12–15). The correlation between cancer and microbiota has become a focal point in oncology research, with remarkable progress has been made revealing the functional roles and therapeutic potential of microbiota in cancer progression (16). Clinically, human papillomavirus vaccination has demonstrated remarkable success in reducing gynecological cancers, such as cervical cancer (17). Meanwhile, *Helicobacter pylori* screening and eradication programs have shown efficacy in gastric cancer (GC) prevention (18). In addition, intratumor microbiota have been implicated in influencing both tumor initiation and metastatic processes (19). These findings have spurred the development of innovative treatment strategies targeting cancer-associated microorganisms, including approaches that modulate microbial communities to enhance therapeutic responses, which is an emerging paradigm with significant clinical potential.

The therapeutic potential of microorganisms against solid tumors was documented firstly over a century ago (20), yet significant progress in this field remained limited until recently. Advances in detection technologies, microbial cultivation methods, and our growing understanding of the TME have now provided compelling evidence of microbial influences on host metabolism and cancer biology, revitalizing research in this area (16). In addition to their individual roles in physiological and pathological processes, the interaction between microbiota and the metabolic reprogramming has emerged as a critical factor in tumorigenesis and therapeutic response (21, 22). For instance, *Akkermansia muciniphila* promotes lung cancer progression by modulating glycolytic, glutaminolytic, and nucleotide metabolism to shape the TME (23). Conversely, certain commensals like *Bifidobacterium pseudolongum*, *Lactobacillus johnsonii*, and *Olsenella* species enhance anti-tumor immunity through inosine-mediated T cell activation (24). Further studies on the crosstalk between metabolic reprogramming and the microorganisms are needed to better examine the correlations between them, and the potential mechanisms by which they influence cancer progression. Therefore, a systematic treatment strategy is urgently needed to effectively identify the current cancer phase and the crosstalk between metabolic reprogramming and the microorganisms, then provide appropriate and effective interventions to discusses their potential for clinical translation to provide new insights into cancer treatment.

In this review, we systematically examine the critical roles of metabolic reprogramming and microorganisms in cancer progression, highlighting their distinct contributions to tumor development. We further analyze how specific bacteria regulate metabolic pathways to influence tumor behavior, as well as how metabolic reprogramming may drive microbiota-mediated carcinogenesis. Our discussion underscores the central role of metabolic reprogramming in mediating the dynamic interplay between microbiota and human tumors. Moreover, we summarized the crosstalk between them and proposed a therapeutic concept based on the theory. That is a set of clinical therapeutic interventions tailored for different cancer stages by targeting the metabolic reprogramming/microbiota axis. In addition, multiple drugs were summarized, and clinical trials or animal experiments were evaluated to assess the therapeutic potential of targeting the metabolic reprogramming/microbiota axis as anticancer strategies.

## Overview of metabolic reprogramming and microbiota

### Metabolic reprogramming and cancer

**Figure 1:**
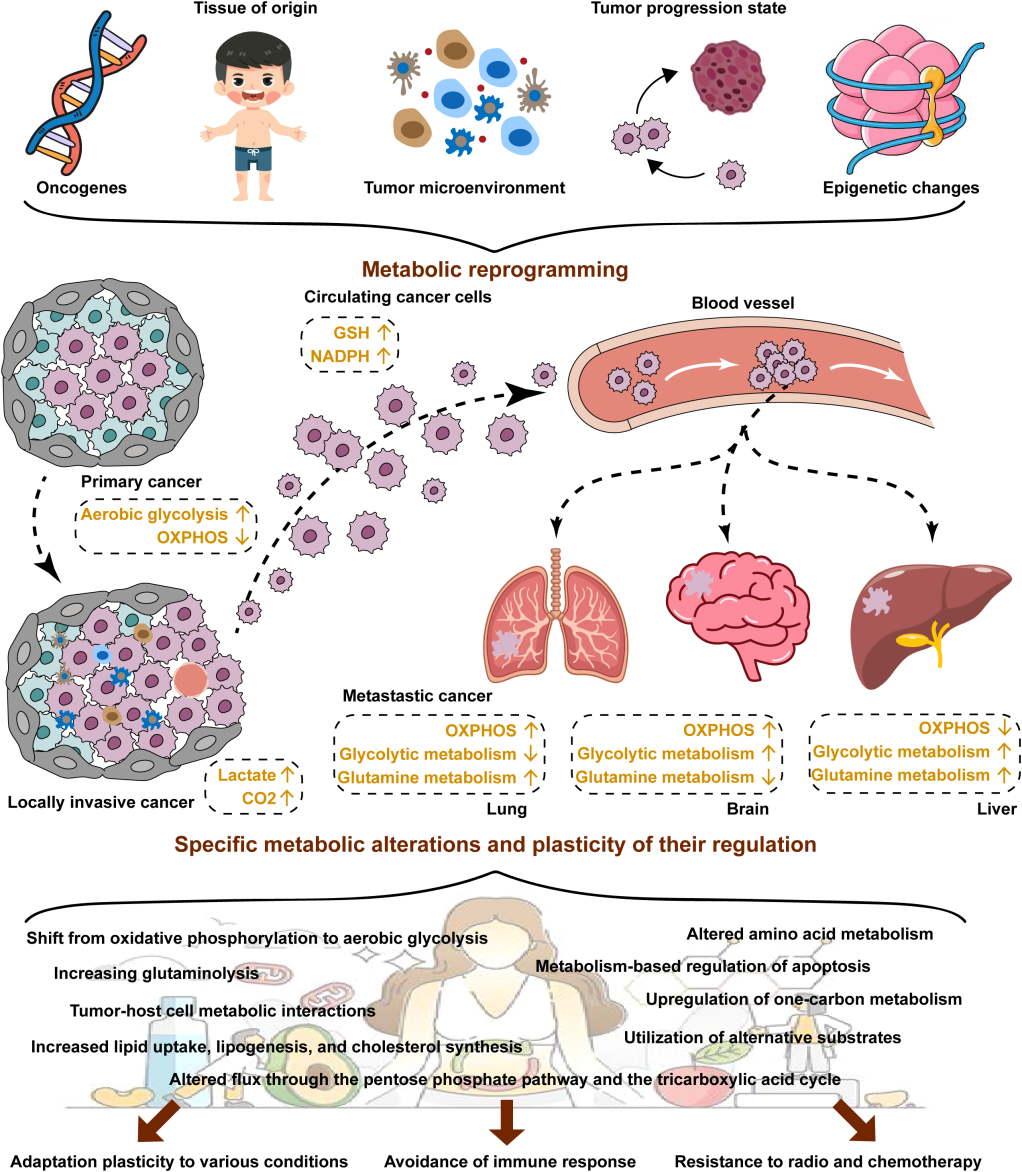
Metabolic reprogramming in cancer. Cancer cells usually exhibit aberrant metabolism resulting from metabolic reprogramming. Metabolic reprogramming is dependent on many factors, including oncogenes, tissue of origins, the TME, tumor progression stage and epigenetic changes. Metabolic reprogramming plays a key role in the reprogramming of adaptation plasticity to various conditions, avoidance of immune response, and resistance to radio and chemotherapy for cancer cells.

Metabolic reprogramming plays a critical role in the maintenance of increased nutrient demands while producing oncometabolites and coping with the demanding the TME (**Figure 1**). Metabolic reprogramming has expanded to cover almost entirely metabolic progressions, including in the metabolism of glucose, fatty acid, and amino acid. This process involves a variety of mechanisms, including regulation of gene expression, the activity of metabolic enzymes, metabolite accumulation and activating signaling pathways (25). In general, there are three main mechanisms in which changes occur: first, the transcriptional level including the activation of transcription factors and the regulation of metabolic pathways by epigenetic modifications (26, 27); second, regulation of metabolic enzyme activities by post-translational modifications, such as phosphorylation and ubiquitination (28, 29); third, changes of metabolites in the TME may also lead to inhibition or activation specific signaling pathways resulting in metabolic reprogramming (30). A large number of studies have found that metabolic reprogramming is closely but complexly related to tumor development, metastasis and drug resistance (4, 25, 31). circRNA circSLIT2 is highly expressed in pancreatic ductal adenocarcinoma and promoted the aerobic glycolysis by targeting miR-510-5p/c-Myc/LDHA axis, ultimately promoting proliferation of cancer cells (32). In colorectal cancer, SIRT1, a hub of metabolic glucolipid conversion, is upregulated and increases the level of deacetylated β-catenin in response to oxidative stress (33). Sodium butyrate can suppress glycolysis by downregulation the expression of HK2 through the c-myc signaling pathway, which results in the suppression of proliferation of the hepatocellular carcinoma (34). Inoue et al. found that pyruvate dehydrogenase (PDH) component X expression is necessary for PDH activity and inhibition of its activity is involved with glycolysis via conversion of pyruvate to lactate, essential for the development of esophageal squamous cell carcinoma (35). Lipids are important components of biological membranes and the type and saturation of fatty acids in the membrane affect its stability and function (8). The key enzyme SCD1 reduces the fatty acid ratio and contributes to the protection of ovarian cancer cells from ferroptosis (36). Lipids are also involved in signal transduction. Prostaglandins (PGs) reduce oxidative stress and prevent lipid peroxidation in tumor cells (37). cPLA2 inhibition shows remarkable synergy restriction to inhibit growth of mutant PIK3CA-bearing breast tumors through influencing the secretion of arachidonic acid (AA) (30). Furthermore, in luminal breast cancer, the mutant of PIK3CA gene initiated the AA metabolic reprogramming through 5-LOX (38). Nelson et al. reported that the USP25/HIF-1α axis is an essential mechanism of metabolic reprogramming and survival in pancreatic ductal adenocarcinoma (39). In addition, the enhanced glycolysis and pentose phosphate pathway were associated with increased HIF-1α expression in colorectal cancer (26). Metabolic phenotypes vary among different types of tumor cells and different stages of tumorigenesis and even in different parts of tumor tissue. Faubert et al. reported that metabolic phenotypes develop as cancer progresses from precancerous lesions to localized, clinically apparent malignancies to metastatic cancer (4). Firstly, metabolic reprogramming can provide malignancy cells with energy and metabolites needed for growth and maintain the stability of the microenvironment (40). Secondly, tumor cells can undergo metabolic reprogramming due to DNA damage, inactivation of genes, and activation of the signaling pathway, thus promoting cancer (41). The above molecules and their mechanisms of regulating metabolic reprogramming are summarized in **Table 1**.

**Table 1:** Representative molecules of metabolic targets.

Cancer type	Targeting molecules	Function	Mechanism	Reference
Pancreatic ductal adenocarcinoma cancer	circSLIT2	Aerobic glycolysis	Via the miR-510-5p/c-Myc/LDHA axis	(32)
	HIF-1α	Glycolysis	Ubiquitin proteasome pathway	(39)
Colorectal carcinoma	SIRT1	Glucolipid metabolic conversion	By upregulating deacetylated β-catenin and translocating it from the nucleus to the cytoplasm	(33)
	HIF-1α	Glucose metabolism	Activating the ROS/PI3K/Akt and Wnt/β-catenin signaling pathways	(26)
Hepatocellular cancer	Hexokinase 2	Aerobic glycolysis	Through the c-myc/hexokinase 2 pathway	(34)
Esophageal squamous cell cancer	PDHX	Tricarboxylic acid cycle	Inhibiting the proliferation of cancer stem cells	(35)
Ovarian cancer	stearoyl-CoA desaturase	Monounsaturated fatty acid synthesis	Ferroptosis	(36)
Breast cancer	cPLA2	Arachidonic acid metabolism	Through the mTORC2-PKCζ axis	(30)
	5-lipoxygenase	Arachidonic acid metabolism	Through the Akt/STAT3 signaling pathway	(38)

### Microbiota and cancer

**Figure 2:**
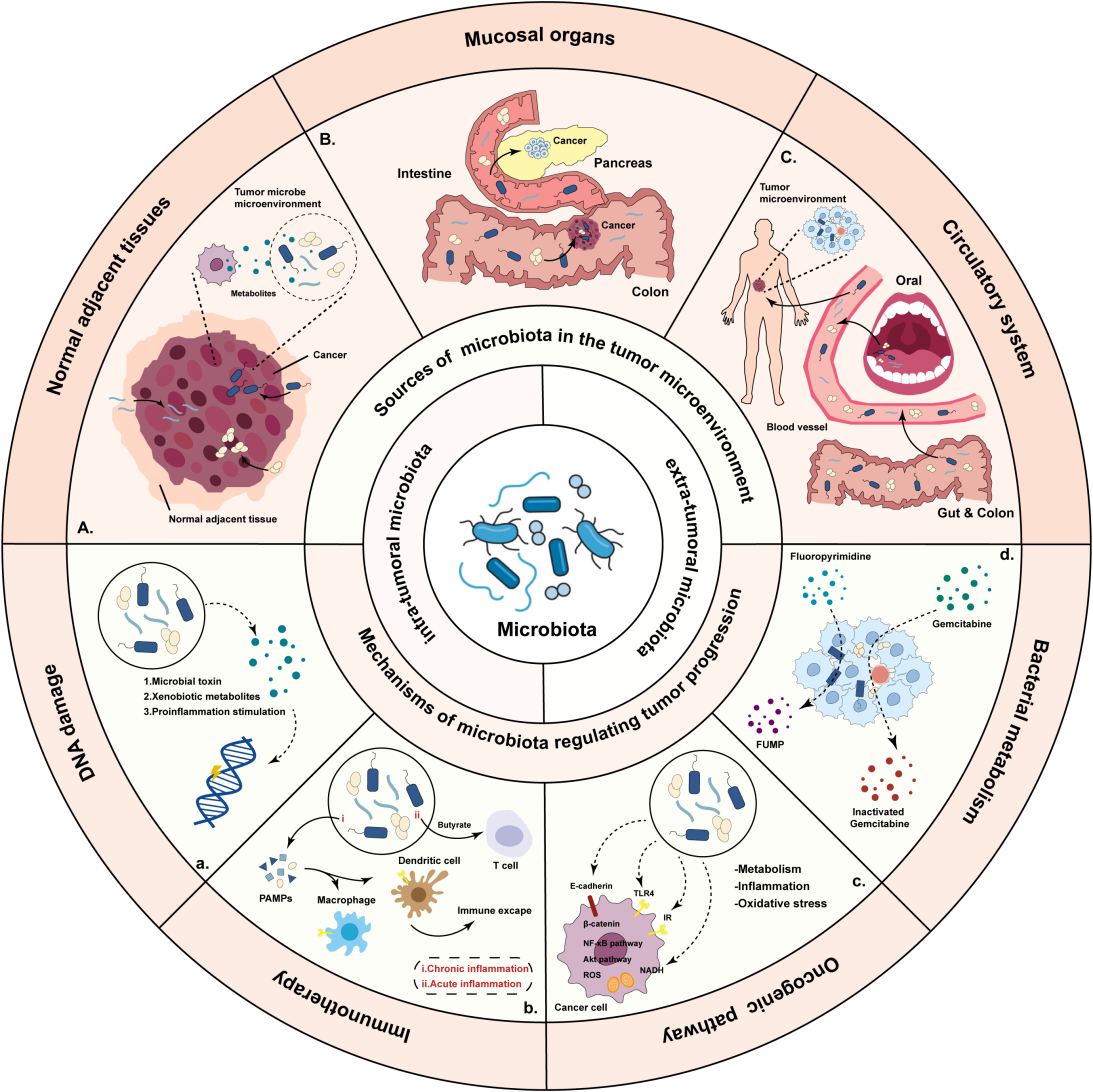
Overview of the sources of the microbiota and mechanisms by which bacteria regulate tumor progression. There are three potential sources of microbiota: microbiota originating from normal adjacent tissues, microbiota from mucosal organs through mucosal barriers, and microbiota which are the result of hematogenous spread. Microbiota influence tumorigenesis and treatment through DNA damage, activating the signaling pathway, influencing the anti-tumor immunity and metabolize drugs.

So far, many important discoveries about microbiota have been reported (**Figure 2**). The microbial community may have an indirect or direct carcinogenic function to regulate cancer initiation, progression and response to therapies by regulation of oncogenic pathways, or modulation of the immune system (42). Bacteria, viruses and fungi are the main microorganisms that regulate promoting mucosal inflammation and human immunity (43–45), where most studies focused on the extra-tumoral microbiota and intra-tumoral microbiota.

Abundant microbes (approximately 4×10^13^ microbial cells) exist and colonize on and inside human (46). The most abundant microorganisms are harbored in the mucosal organs of human bodies, including in the intestinal tract, oral cavity, and skin (11, 16). The microbiota and the mucosal organs form a symbiotic, holistic system together, and maintain the homeostasis as a biological barrier. Interestingly, the mice which are bred and housed in an environment devoid of microorganisms were immune deficient and exhibited a flimsy gut barrier (47). Indeed, studies have shown that pathogenic bacteria in mucosal organs are associated with multiple cancers, such as head and neck cancer, lung cancer, colorectal cancer, esophageal cancer, and pancreatic cancer (48–51). The progression of skin cancer has been reported to be related to bacteria through the TLR-5 signaling pathway (52). *Fusobacterium nucleatum* might utilize the TLR4/Keap1/NRF2 signaling to promote colorectal cancer development and metastasis (53). The detection rate of *Streptococcus* and *Clostridium* in gastric cancer are higher than those in normal tissue, whereas *Lactobacillus brevis* is more enriched in normal controls (54). Shi et al. reported that microbial richness is significantly decreased in gastric cancer tissues compared with adjacent normal tissues, and some microbes, such as *Cupriavidus* and *Sphingomonas*, are enriched in cancer tissues, while *Ochrobactrum* are enriched in normal tissues (55). In addition, the mycobiome also plays an important role in tumorigenesis of pancreatic ductal adenocarcinoma. The cancer tissues displayed an increase in fungi of about 3,000-fold compared to normal pancreatic tissue, and the *Malassezia* sp*ecies* were found to be associated with oncogenesis (56). Researchers also found *Porphyromonas gingivalis*, which is highly epidemically connected with pancreatic cancer, promoted pancreatic cancer progression via elevating the secretion of neutrophilic chemokines and neutrophil elastase (57).

The tumor tissues once considered sterile before, however, with the development of technology, researchers actually discovered a variety of microorganisms are also found in tumors that do not arise from mucosal sites (58, 59). Therefore, the concept of intra-tumoral microbiota present in tumor tissues is proposed (60), and intra-tumoral microorganisms have been found in at least 33 major cancer types, such as breast cancer (61, 62). Certain bacteria in the breast tissues are associated with cell dysplasia and carcinogenic effects. The levels of *Pseudomonas*, *Porphyromonas*, and *Proteus* in breast cancer tissues are significantly higher than those in normal tissue, while *Propionbacterium* and *Staphylococcus* are less enriched than that in the non-tumor tissue conversely (62). *Enterotoxigenic Bacteroides fragilis* have been shown to be cancer-causing bacteria in breast cancer, it activates the notch and beta-catenin axes and induces growth and metastasis (63). Furthermore, *Faecalibacterium prausnitzii* is less abundant in breast cancer patients, which could suppress the growth of breast cancer cells through inhibition the JAK2/STAT3 signaling pathway (64). Recent studies have revealed that *H. pylori*‐NF‐κB activates the PIEZO1‐YAP1‐CTGF axis to remodel the GC microenvironment by promoting CAF infiltration (65). The detection rate of *Aquificae* and *Planctomycetes* in ovarian carcinoma is higher than that in adjacent tissue, and the level of *Crenarchaeota* is decreased (66). Researchers also found that the development of ovarian carcinoma is related to *Chlamydia*, *Mycoplasma*, *Acinetobacter*, and *Brucella (* 67). A recent study analyzed the composition of intra-tumoral microbiota in pituitary neuroendocrine tumors. In this study, researchers found that *Fusobacteriaceae*, *Tissierellaceae*, *Aerococcaceae*, and *Corynebacteriaceae* may correlate with the pathogenesis and development of the tumors (68). Nejman et al. reported that microbiota (such as *Fusobacteriaceae*, *Tissierellaceae*, *Aerococcaceae*, and *Clostridiales*) was detected in tumors that have no direct connection with the external environment, such as glioblastoma multiforme and bone cancer (58). The abovementioned bacteria and other microbiota in a few cancers are all summarized to understand their role cancer progression (**Table 2**).

**Table 2:** Microbial heterogeneity in different tumors.

Cancer type	Microorganisms	Quantitative dynamics	Tumor behavior	Mechanism	Reference
Colorectal cancer	*Fusobacterium nucleatum*	Increase	Proliferation		(51)
	*Bifidobacterium*	Increase	Facilitate immunotherapy		(49)
	*Fusobacterium Nucleatum*	Increase	Invasion and migration	Activating a Cytochrome P450/Epoxyoctadecenoic Acid Axis via TLR4/Keap1/NRF2 Signaling	(53)
Gastric Cancer	*Peptostreptococcus, Streptococcus, and Fusobacterium*	Increase	Enrichment in tumor tissues		(54)
	*Lactococcus lactis and Lactobacillus brevis*	Decrease	Enrichment in non-tumor tissues		(54)
	*Helicobacter pylori*	Increase	Carcinogenesis		(54)
	*Cupriavidus and Sphingomonas*	Increase	Enrichment in tumor tissues		(55)
	*Helicobacter pylori*	Increase	Progression and peritoneal metastasis	By activating the PIEZO1-YAP1-CTGF axis and remodeling the microenvironment	(65)
	*Ochrobactrum*	Decrease	Enrichment in non-tumor tissues		(55)
Pancreatic ductal adenocarcinoma	*Malassezia* spp	Increase	Carcinogenesis	Ligation of mannose-binding lectin	(56)
Pancreatic cancer	*P. gingivalis*	Increase	Promoted the tumor progression	Shapes the immune system	(57)
Breast cancer	*Pseudomonas, Porphyromonas, and Proteus*	Increase	Carcinogenesis		(62)
	*Propionbacterium and Staphylococcus*	Decrease	Enrichment in non-tumor tissues		(62)
	*Bacteroides fragilis*	Increase	Tumor growth and metastasis	Activating the Notch and β-Catenin Axes	(63)
	*Faecalibacterium prausnitzii*	Decrease	Aapoptosis	Inhibit the secretion of IL-6 and the JAK2/STAT3 pathway	(64)
	*Firmicutes and Bacteroidetes*	Decrease	Enrichment in non-tumor tissues	Metabolic reprogramming	(64)
	*Verrucomicrobla, proteobacteria and actinobacteria*	Increase	Enrichment in tumor tissues	Metabolic reprogramming	(64)
Ovarian cancer	*Aquificae and Planctomycetes*	Increase	Enrichment in tumor tissues		(66)
	*Crenarchaeota*	Decrease	Enrichment in non-tumor tissues		(66)
	*Chlamydia, Mycoplasma, Acinetobacter, and Brucella*	Increase	Enrichment in tumor tissues		(67)
Pituitary neuroendocrine tumor	*Fusobacteriaceae, Tissierellaceae, Aerococcaceae, and Corynebacteriaceae*	Increase	Carcinogenesis		(68)
Glioblastoma	*Fusobacteriaceae, Tissierellaceae, Aerococcaceae, and Clostridiales*	Increase	Carcinogenesis		(58)
Bone cancer	*Fusobacteriaceae, Tissierellaceae, Aerococcaceae, and Clostridiales*	Increase	Carcinogenesis		(58)

## Crosstalk between metabolic reprogramming and microbiota in cancer development

**Figure 3:**
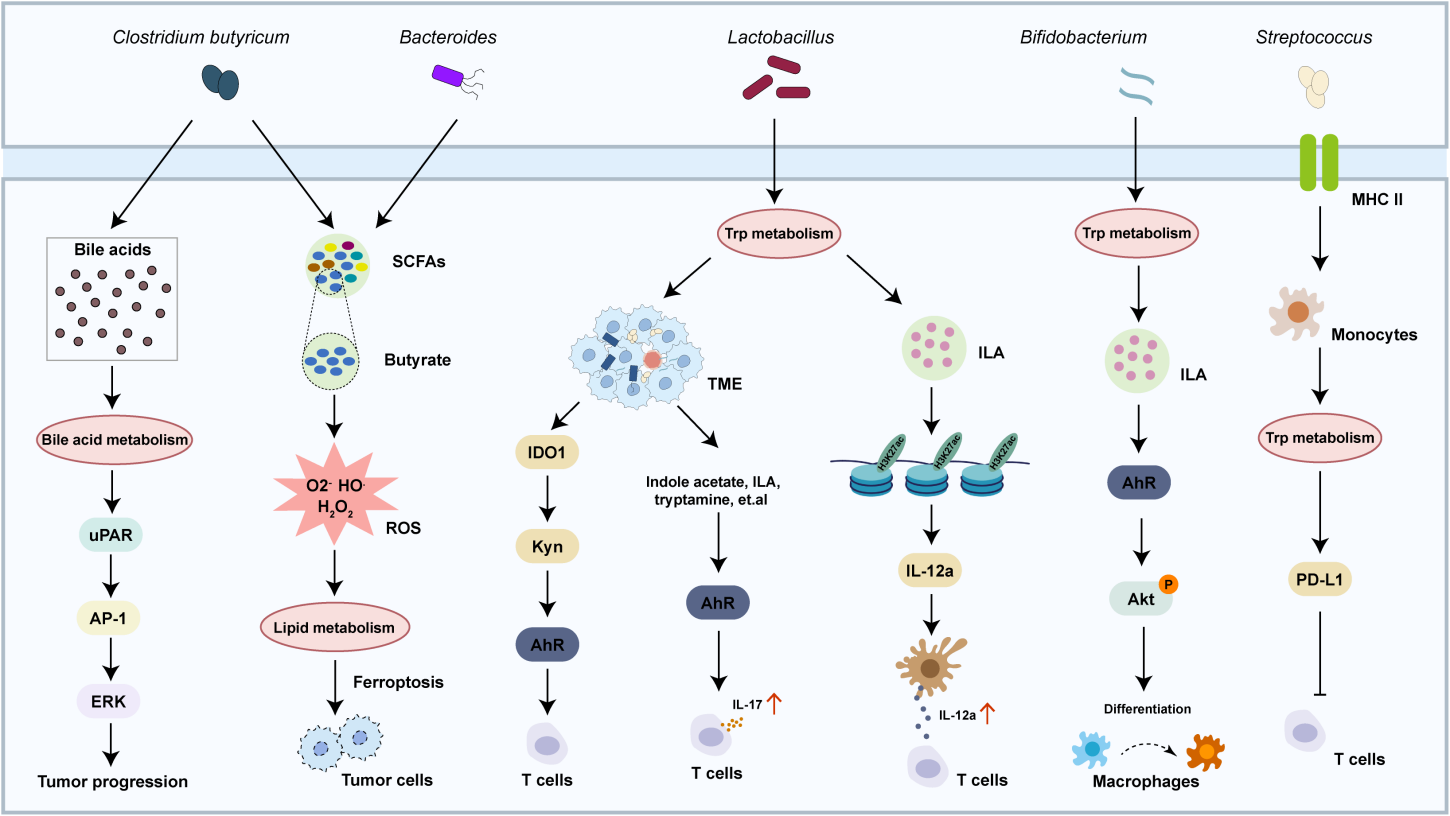
Crosstalk between metabolism pathway and microbiota in cancer. The crosstalk between metabolism pathway and microbiota regulates multiple physiological and pathological responses, including cancer progression and reshaping the TME.

**Figure 4:**
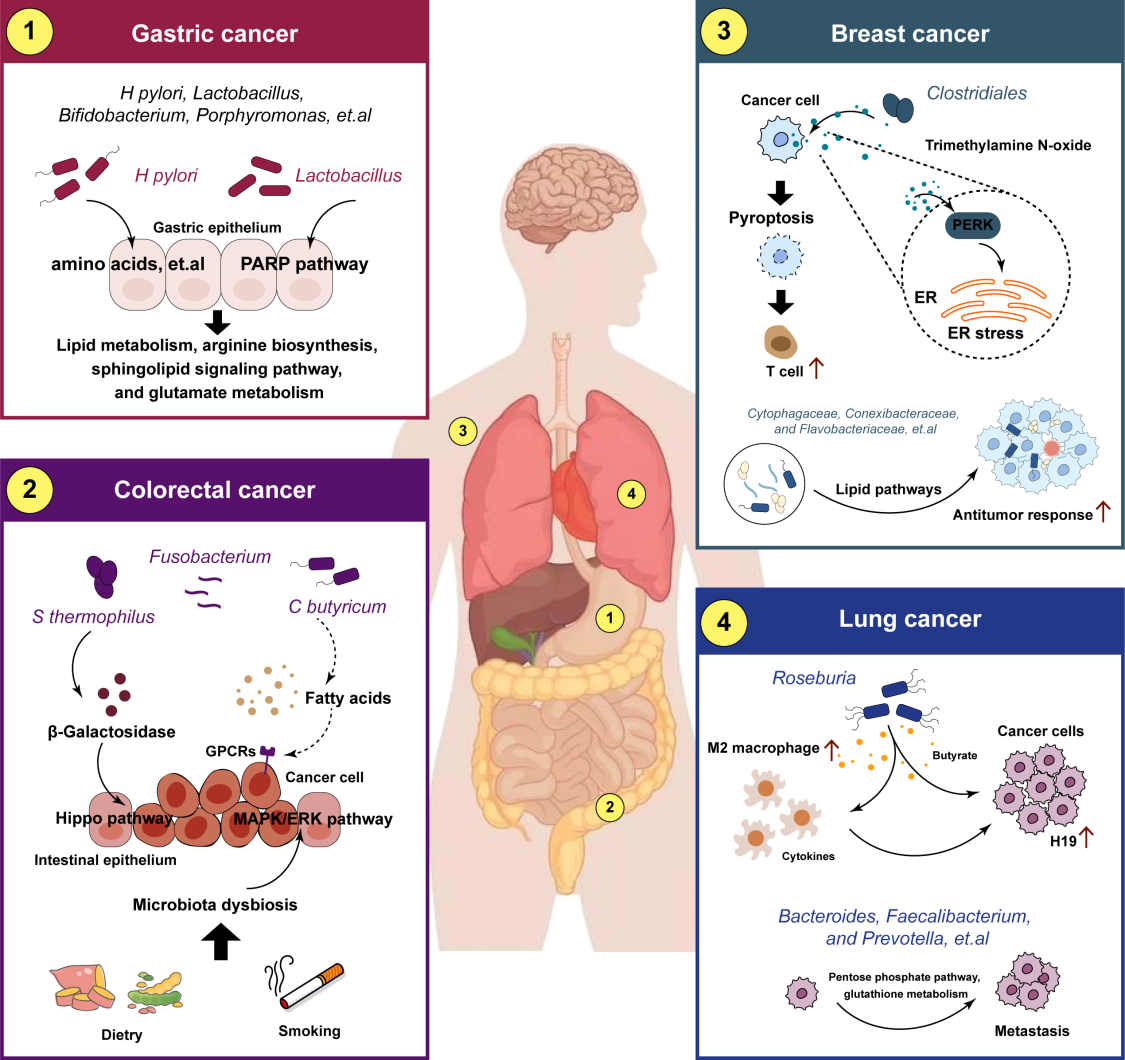
Crosstalk between metabolic reprogramming and microbiota in different tumors. The crosstalk between metabolic reprogramming and microbiota regulates cancer progression. metabolic reprogramming can play a role in microbiota-mediated tumorigenesis, metastasis and drug resistance in different cancers.

Specific microbiota intricately regulates tumor progression through dynamic interactions with the metabolic pathways in the host through three key mechanisms: regulation the metabolism of cancer cells, nutrient competition with immune cells and reshaping the TME, and immune-metabolic reprogramming (69–71) (**Figure 3**). Commensal bacteria like *Clostridium butyricum* and *Bacteroides* ferment dietary fiber to produce short-chain fatty acids (SCFAs), where butyrate triggered superoxidative stress and intracellular lipid accumulation, which enhanced ferroptosis susceptibility in cancer cells (72, 73). Concurrently, species such as *Clostridium* transform primary bile acids into tumor-promoting secondary bile acids that activate the expression of urokinase-type plasminogen activator receptor (uPAR) to drive ERK and AP-1 signaling in colon cancer cells (74). The TME is further shaped through microbial nutrient metabolism, where tryptophan metabolism by *Lactobacillus* activates the aryl hydrocarbon receptor to enhanced T cell production of IL-17 (75), while *Bifidobacterium* breve-derived indole-3-lactic acid (ILA) ameliorates tumorigenesis by directing the differentiation of macrophages (76). Microbiota also orchestrate immune-metabolic crosstalk, with streptococcal pyrogenic exotoxin A (SPEA) of *Streptococcus* promoting CD25^+^/Foxp3^+^ Treg expansion via PD-L1 and kynurenine (77), whereas *Lactobacillus gallinarum*-derived metabolites inhibit the function of Tregs through modulating IDO1/Kyn/AhR axis (78). Epigenetic modifications, including *Lactobacillus plantarum* and its metabolite enhancing H3K27ac binding at the enhancer regions of IL-12a, further priming CD8^+^ T cell immunity against tumor growth (79). These findings highlight the microbiome as a master regulator of tumor metabolism, offering novel avenues for intercepting cancer progression through microbial-metabolic reprogramming. The crosstalk between metabolic reprogramming and microbiota varies substantially across different types and stages of malignancy. Here, we summarized the microbiota in a few cancers to understand the role in cancer development (**Figure 4**).

### Gastric cancer

The gastric microbiota and their metabolites are the major risk factors for the development and progression of gastric cancer (GC). *Helicobacter pylori* is the most predominant microorganism detected in GC, and it has been associated with precancerous lesions that can eventually promote the development of GC (80). The specific mechanisms by which *H. pylori* mediate the carcinogenesis and progression of GC are still unclear, and is associated with multiple factors, including in inflammatory responses, host genetic diversity, and environmental influences (11). Recent studies have revealed that the reason for the differences in metabolome profiles between GC tumor and non-tumor tissues may due to the collective activities of *H. pylori*, *Lactobacillus*, and other microorganisms (81). The metabolome analysis demonstrated that *H. pylori* is negatively and positively correlated with the majority of differential metabolites in the classes of amino acids, carbohydrates, nucleosides, nucleotides, and glycerophospholipids, respectively, suggesting that it might play a key role in degradation and synthesis of the majority of differential metabolites (82). Moreover, alterations in gut microbiota and metabolism are potentially linked to chronic inflammation and GC (83). In precancerous lesions of GC rat model, *Lactobacillus* and *Bifidobacterium* increased significantly while *Turicibacter* and *Romboutsia* reduced significantly, mechanically, the microbiota and the metabolites are related to the lipid metabolism and PPAR signaling pathway (84). Additionally, Wang et al. reported that Lactobacillus could enhance the production of N-nitroso compounds, thus, these enriched bacteria could participate in the carcinogenesis (85). Yang et al. observed that *Methylobacterium-Methylorubrum* was significantly increased in distal gastric cancer tissues, positively correlated with cancer-promoting metabolites, including in arginine biosynthesis, sphingolipid signaling pathway, and glutamate metabolism (86). Meanwhile, *Porphyromonas*, *Catonella*, *Proteus*, *Oribacterium*, and *Moraxella* were significantly correlated with hormone metabolism in proximal gastric cancer (86).

### Colorectal cancer

Metabolic reprogramming are important components in the crosstalk between the gut microbiota and the human body and play key roles in the development of colorectal cancer (CRC) (87). Mechanistically, microorganisms produce metabolites after the uptake of certain nutrients, which include lipids, proteins, secondary bile products, biogenic amines, oligosaccharides, glycolipids, organic acids, and amino acids, and then the metabolites act as signaling elements and substrates in metabolic reactions (88, 89). Metabolic reprogramming serves vary functions in the progression of CRC, and further studies have shown that metabolic reprogramming plays a dual role (90). Coker et al. demonstrated that gut metabolites and their association with amino acids metabolic pathways in the colorectum are altered in the early the stages leading to CRC (91). Bile acid was recently been demonstrated to be bio-transformed by the microbiota and play a role in the microenvironment in Inflammatory bowel disease and CRC (92). *Clostridium Butyricum* is one of the original gut microbiota species found in human. *C. butyricum* can produce SCFAs, which can directly activate G-coupled-receptors, inhibit histone deacetylases, and serve as a protector in CRC (93). Li ert.al verified that *Streptococcus Thermophilus* could secret β-Galactosidase to activate oxidative phosphorylation and downregulate the Hippo signaling pathway, which lead to the suppressive effects of CRC cells (94). *Fusobacterium nucleatum* enrichment and short-chain fatty acid depletion characterizes late-onset CRC, while early-onset CRC tended to be associated with increased tryptophan, bile acid and choline metabolism and enriched *Flavonifractor plauti (* 95). Furthermore, high-fat diet drives CRC through modulating microbiota and metabolites (96). Yang et al. reported that the increased bacteria of *Alistipessp.Marseille-P5997* and *Alistipessp.5CPEGH6*, and depleted probiotic *Parabacteroides distasonis*, along with elevated lysophosphatidic acid in CRC mice model (97). In addition, the smoke-induced gut microbiota dysbiosis, including the enrichment of *Eggerthella lenta* and depletion of *Parabacteroides distasonis* and *Lactobacillus* spp, increased the bile acid metabolism and impaired gut barrier function, through activating the MAPK/ERK signaling pathway in CRC cells (98).

### Breast cancer

Breast cancer (BC) is the most widespread malignant cancer among women worldwide and presents significant challenges to female health (99). The microorganisms exert a profound impact on the host and has emerged as a pivotal frontier in the BC pathogenesis (100). In recent years, a growing number of scholars have reported that BC tissues have a great diversity and abundance of microorganisms (58). Hieken et al. discovered that the differences of *Lactobacillus* between the breast tissue in benign and malignancy, which are associated with metabolic pathway involving cysteine and methionine metabolism, glycosyltransferases, fatty acid biosynthesis (101). Xuan et al. demonstrated that genus *Cytophagaceae*, *Conexibacteraceae*, and *Flavobacteriaceae* possibly modulate the BC immune microenvironment and elicit an antitumor response through lipid pathways (102). Another study revealed that metabolite of *Clostridiales*, trimethylamine N-oxide, induced pyroptosis by activating the endoplasmic reticulum stress kinase PERK in triple-negative BC (103). Heath et al. reported that gut microbiota-derived metabolites alter estrogen receptor activity and endocrine therapy responsiveness in ER^+^ BC, and targeting metabolic pathways through diet or drugs could be useful to improve endocrine therapy efficacy in the clinic (104). Interestingly, women with malignant breast carcinoma are found to exhibit enriched level of *Citrobacter* in their gut, which is associated with elevated glycan and lipopolysaccharide biosynthesis, in comparison to the group with benign tumors (105). One of the pivotal independent risk factor for breast tumorigenesis is hormonal deregulation, and multiple studies have shown a direct role of gut microbiota in hormonal deregulation through influence the expression of β-glucuronidase (100). Furthermore, indicated the relationship between microorganisms and metabolism, and found their potential roles in the prognosis value, indicating that patients with both high Campylobacter abundance and inositol phosphate metabolic activity had the worst survival probability (106).

### Lung cancer

The lung is another organ with an abundant microbiome, which leads to the exposure of lung cancer to numerous microorganisms (107). It is certain that the composition of lung microbiota is altered in lung cancers, which promoted tumor growth and metastatic progression (16). Smoking is the most important environmental risk factor associated with lung cancer (108). A recent study analyzed the correlation of microbes and smoking‐related metabolic pathways (109). In this study, researchers found that *Acidovorax* is enriched in smokers. Additionally, Ma et al. found that *Roseburia*-derived butyrate promotes lung cancer metastasis by increasing expression of H19 in tumor cells through inhibiting HDAC2 and increasing H3K27 acetylation at the H19 promoter and inducing M2 macrophage polarization (110). Notably, deep microbiome sequencing and targeted bacterial culture confirmed that the tumor-resident *Lactobacillus* in the TME in lung cancers can alter tumor metabolism and lactate signaling pathways (111). A retrospective study showed that the characteristics of the microbiome and metabolite presented significantly differences between lung cancer patients and benign pulmonary nodules patients (112). The abundance of *Subdoligranulum* and *Romboutsia* increased in lung cancer patients, combined with the enrichment in beta-Alanine metabolism, styrene degradation and pyrimidine metabolism pathway. Liu et al. found that the microbiome of lung cancer patients, such as *Enterobacteriaceae* and *Streptococcus*, had increased expression of gene modules involved in metabolism and amino acid metabolism (113). Microorganisms have been shown to participate in the metabolism of bile acids and proteins and to help form aromatic amines and sulfides, subsequently promoting lung cancer progression (114). Moreover, microbial metabolites were shown to be significantly altered in lung cancer (115). Recently, Wang and colleagues reported evidence that *Bacteroides*, *Faecalibacterium*, and *Prevotella* may participate in regulating metabolism-related pathways, such as the pentose phosphate pathway and glutathione metabolism, in lung adenocarcinoma (116).

### Pancreatic cancer

The incidence of and number of deaths caused by pancreatic cancer have been gradually rising, and it is a leading cause of cancer death worldwide (117). The role of microbiota and metabolic reprogramming in pancreatic cancers have been proven in several studies. Tintelnot et al. reported that the tryptophan metabolite, 3-IAA, derived from microbiota as a key amplifier of the response to chemotherapy in pancreatic ductal adenocarcinoma (PDAC) (118). Furthermore, a recent study showed that *Lactobacillus* participate in the process that tryptophan-derived metabolites activate the aryl hydrocarbon receptor in tumor-associated macrophages to suppress immunotherapy (119). Alam et al. discover that intratumor mycobiome, such as *Malassezia* and *Alternaria*, activates the secretion of IL-33, which can induce metabolic reprogramming and accelerate the development of PDAC (45). High-fat diet has been confirmed as one of a risk factor for the development of pancreatic cancer (120). The disturbance of lipid metabolism could induce changes the intestinal environment and further leading to the dysbiosis of internal microflora in mice model of pancreatic cancer (121). Ruze and colleagues reported that the main mechanisms involved in the pancreatic carcinogenesis include microbiome dysfunction further compromise immunometabolic regulation while also aggravating mutagenic and carcinogenic metabolic disorders by affecting multiple pathways (122).

### Other cancers

In addition to the cancers reported above, multiple studies have also shown the crosstalk between microbiota and metabolic reprogramming in other tumors. A clinical study showed that the existence of microorganisms in the tumor tissue of head and neck squamous cell carcinoma (HNSCC) patients (123). Sun et al. reported that *F. nucleatum* affects the tumor immune microenvironment via modulating via GLUT1-driven glycolysis and extracellular lactate deposition (124). This is consistent with a study by Colbert et al. showed that lactic acid bacteria in the TME can alter tumor metabolism and lactate signaling pathways, affecting chemoradiation response in patients with cervical cancer (111). Yost et al. showed that the microorganisms of OSCC patients are associated with the increased activities of iron ion transport-related enzymes, tryptophanase, glutamate dehydrogenase, starch synthase, and superoxide dismutase (125). Additionally, recent studies have demonstrated that microbiota, which drastically alter the metabolome of cervical cancer, is involved in HPV persistence, progression of cervical neoplasia, and genital inflammation (126). Ilhan and colleagues showed that cervicovaginal metabolic profiles, charactered by amino acid and nucleotide metabolisms, were driven by genital inflammation, HPV infection, and vaginal microbiota, including *Sneathia*, *Streptococcus*, *Prevotella*, and *Gardnerella (* 127). 16S rRNA gene sequencing and untargeted metabolomics revealed that *Prevotella* is involved in the synthesis of fatty acyl, carboxylic acids and derivatives, benzenes and substituted derivatives, organic oxygenates, and indoles and derivatives as metabolites. *Fusicatenibacter* and *Lachnospira* are involved in the degradation of indoles and derivatives. *Alistipes*, *Agathobacter*, and *Parabacteroides* are involved in the synthesis of indoles in esophageal squamous cell carcinoma (ESCC) (128). Further analysis revealed that *Prevotella*, *Alistipes*, *Agathobacter*, and *Parabacteroides* might regulate the synthesis of indoles and promote ESCC. Cheung et al. observed that an enrichment of carcinogenic bacteria, such as *Butyricimonas*, *Veillonella*, and *Streptococcus*, and a depletion of *Butyricicoccus* of ESCC patients (129). Recently, Lau et al. reported that *L. acidophilus* exhibits anti-tumorigenic effect in mice by secreting valeric acid, and probiotic supplementation is a potential prophylactic of hepatocellular carcinoma (130). The relationships between metabolic reprogramming and microorganisms of cancers have not been thoroughly studied.

## Targeting metabolism/microbiota as an emerging therapy in cancer

Since metabolism plays an important role in the development of human malignancy, it may promote tumor formation, or inhibit tumor cell growth. Alternatively, the abovementioned microorganisms may cause cancer through certain mechanisms. In recent years, breakthrough results have been obtained in clinical trials assessing the relationship between metabolic reprogramming and microorganisms in cancer. In the following section, we summarized the drugs, as well as the corresponding clinical trials, that target metabolic reprogramming or microbiota and exert anticancer effects and their mechanisms. More importantly, we emphasized the critical role of metabolism reprogramming in mediating microbial communities and cancer. A variety of bacteria regulate tumor development, metastasis and treatment-resistant through metabolic reprogramming. Therefore, the simultaneous use of drugs targeting metabolism and microorganisms may play a synergistic anticancer role. However, few such studies have been performed, and more clinical trials are urgently needed to better understand the therapeutic potential of drugs targeting the metabolic reprogramming/microbiota axis, which could help to develop new drugs to prevent or treat human cancer.

## Targeting metabolism for cancer treatment

Metabolic reprogramming is not only a biological hallmark of cancer but also reveals treatment vulnerabilities. The vulnerability caused by metabolism rewiring may present therapeutic opportunities as malignancy cells become more and more dependent on specific metabolic pathways. Farber and Diamond reported that the anti-folate drug, aminopterin, could induce remission in pediatric acute lymphocytic leukemia as early as 1948, and has been used as an anti-tumor therapy for many years (131). Xiao Y. indicated that the metabolic targets for cancer treatment could be categorized into three groups: (i) targeting the metabolic vulnerability of tumor cells, (ii) targeting metabolism of the TME, and (iii) regulating body metabolism (132). Considering the active nucleotide synthesis and high proliferation rate of cancer cells, disrupting nucleotide metabolism is considered a promising anti-tumor therapeutic strategy (133). Except the aminopterin, many purine and pyrimidine analogs have been widely used in the clinic, including in 5-fluorouracil, 6-mercaptopurine, gemcitabine, capecitabine, and fludarabine (132, 134, 135). Jin et al. reported that the treatment of leflunomide, a drug that inhibits dihydroorotate dehydrogenase, prevented lung metastasis in the mouse lung cancer metastasis model (136). Studies have shown that inhibitors of dihydroorotate dehydrogenase can exhibit antitumor effects in a variety of preclinical models, such as glioma (137, 138) and lymphoma (139). Furthermore, studies have shown that some promising metabolic targets in energy metabolism for cancer treatment, including in glycolysis, fatty acid metabolism, glutamine metabolism, the tricarboxylic acid (TCA) cycle, and oxidative phosphorylation (OXPHOS) (140, 141). Tran et al. reported that α-Ketoglutarate promote stemness and leads to CRC formation through Wnt signaling pathway (142). Inhibitors targeting glucose transporters (GLUT), hexokinase, lactate dehydrogenase, and lactate-proton symporters have shown promise in impairing tumor growth (132). Additionally, enzymes involved in fatty acid oxidation (FAO) and fatty acid synthesis and desaturation are being actively studied (143). Inhibitors of glutamine transporters and glutaminases have also been investigated (144). Han et al. demonstrated that activation of polyamine catabolism promotes glutamine metabolism and creates a targetable vulnerability in lung cancer cells (145). A phase I clinical trial and pharmacodynamic study of complex I inhibitor of oxidative phosphorylation in advanced solid tumors and acute myeloid leukemia patients showed that strategies targeting the TCA cycle and OXPHOS, such as inhibitors targeting electron transport chain (ETC) complexes, have displayed antitumor effects (146). Metformin, a drug known to inhibit ETC complex I, has been extensively studied in clinical trials for cancer treatment (147). Moreover, except for tumor cells, immune cells also have metabolic reprogramming during tumor progression to facilitate the escape of tumor cells from immune surveillance (148, 149). Myeloid cells play a pivotal role in tumor biology, and they play key roles on tumor growth and antitumor immune responses (150). Geiger et al. reported that L-arginine concentrations directly impact the metabolic fitness and survival capacity of T cells, which are crucial for anti-tumor responses (151). In addition, the metabolic reprogramming also could happen in the stromal cells, such as cancer-associated fibroblasts (CAFs). CAFs require proline synthesis by PYCR1 for the deposition of pro-tumorigenic extracellular matrix (152). Beyond metabolic reprogramming at the tumor site, the metabolism of the whole body also presents as a potential therapeutic target. Physical exercise and dietary interventions are the two most important and easily controlled factor that can reduce cancer progression (153, 154). For instance, cPLA2 inhibition shows remarkable synergy with dietary fat restriction to restore tumoral immune cell infiltration and inhibit growth of mutant PIK3CA-bearing breast tumors (30).

## Targeting microorganisms for cancer treatment

Traditionally, some specific microorganisms may act as direct factors responsible for the development of cancer (61). However, the survival benefits of tumor bacteria on tumor cells are most prominent during metastasis rather than primary tumor growth in recent studies (155). Antibiotics have been used clinically to eradicate microorganisms to prevent cancer as much as possible and to assist in the treatment of cancer. For instance, the eradication of *H. pylori* may inhibit the occurrence of gastric cancer (156). Additionally, a retrospective investigation found that antibiotic therapy improves the survival of pancreatic adenocarcinoma patients (157). Recent studies demonstrated that reduction of *Staphylococcus* in the mammary tumor microbiota induces antitumor immunity and decreases breast cancer aggressiveness in mice models (158). Another mouse study indicated that a direct influence of long-term ampicillin exposure can alter the lung microbiota of rats (159). In addition, the treatment of mice bearing a colon cancer xenograft with the antibiotic metronidazole reduced *Fusobacterium* load, cancer cell proliferation, and overall tumor growth (160). Furthermore, some studies indicated that the use of antibiotics can disturb the balance of the gut microbiome, which further might ultimately lead to an unpredictable array of consequences (161). A study indicated that wide-spectrum antibiotics treatment prior to CD19-targeted chimeric antigen receptor T-cell immunotherapy (CAR-T) is associated with adverse outcomes for B cell lymphoma patients (162). In addition to antibiotics treatment, using microorganisms to active anti-tumor immune response and using bacteria as carriers that can release drugs are the main frontier in the treatment of malignancy (16). One example is the use of the BCG vaccine, which is one of the most successful immunotherapies in oncology to date (163). Treatment with BCG is effective for many cancers in clinical therapies, especially bladder cancer (164, 165). Routy et al. demonstrated that fecal microbiota transplant and sufficient dietary fiber intake could improve immunotherapy response rates, and was associated with improved progression-free survival of melanoma patients (166). Besides, some microbiota such as *Listeria* and *Salmonella typhimurium* are used as delivery platforms for drug targeting (167–169). Furthermore, Wu and the colleagues constructed a bacteria-inspired microbots for the treatment of metastatic triple negative breast cancer (170). Recently, a group of researchers developed an engineered probiotic *Escherichia coli* that improved the anti‐tumor effect (171). We summarized representative clinical trials that focused on metabolism and microbiota (**Table 3**). Together, therapies based on microorganisms have attracted a lot of attention.

**Table 3:** Representative clinical trials targeting metabolism and microbiota.

Cancer type	Arms and interventions	Outcome measures	Trial ID
Colorectal cancer	Dietary intervention	Changes in inflammatory markers and microbial metabolites.	NCT06603519
Colorectal cancer	Dietary intervention	Bile acid metabolism and gut microbiome.	NCT04753359
Prostate cancer	Observation	Metabolic characteristics in the prostate tissue in men with different gut microbiota signatures.	NCT06116851
Melanoma, lung cancer, or breast cancer	MetfOrmin	Glucocorticoid-induced diabetes and other metabolic perturbations on gut microbiota populations.	NCT04001725
Head and Neck Squamous Cell Carcinoma	Observation	Trp pathway and oral dysbiosis in human HNSCC cases.	NCT05837221
Colorectal cancer	PD-1 inhibitors	Effect of gut microbiota and its metabolites on the efficacy of immunotherapy in metastatic colorectal cancer.	NCT06714903
Oesophageal cancer	Observation	Profiling microbiome associated metabolic pathways.	NCT06302660
Colorectal adenocarcinoma	Antibiotic administration or probiotic oral intake	Overall survival.	NCT03843905
Skin cancer	Immunotherapy	The relationship between microbes and metabolism during skin cancer immunotherapy.	NCT03370861
Non-small cell lung carcinoma	Immune checkpoints inhibitors	Link metabolic signature with microbiota composition and immune profile.	NCT04189679
Gastric cancer	Observation	Explore the differences in microbial and metabolic characteristics for early screening of gastric cancer.	NCT05812287
Colorectal cancer and pancreaticobiliary cancer	Observation	Analysis for microbiota and metabolism on gastrointestinal neoplasms.	NCT04363983

## Antitumor effects of microorganisms combined with metabolic targets

**Figure 5:**
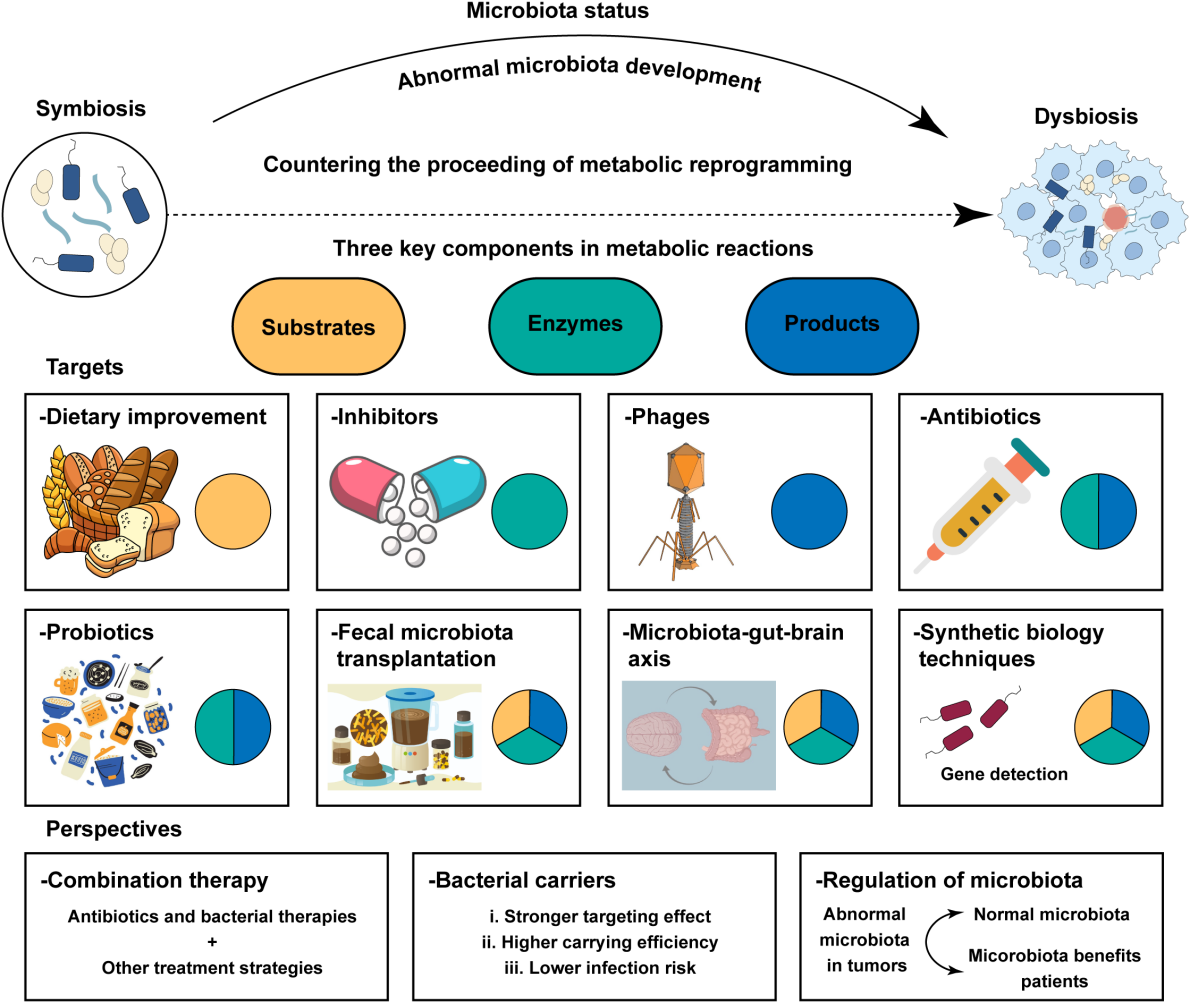
Treatment strategies and prospect of clinical application based on the crosstalk between metabolic reprogramming and microbiota. Metabolic reactions imply three components: substrates, enzymes, and products. The regulation and alteration of each component will affect the metabolic process and have an impact on cancer treatment. Potential strategies enhance the efficiency of anti‐tumor therapies including in researching better bacterial carries, normalizing microbiota in tumor patients, and bacterial therapies combined with other anti‐tumor therapies.

At present, there are few antitumor studies evaluating the effect of combined therapy with antibiotics and metabolic targets, and most current studies are based on gut microbes. It remains unknown whether changing the gut microbes will affect the characteristics of the host metabolism and the progression of cancer; these aspects need to be explored. Gut microbiota‐derived metabolites are key hubs connecting the gut microbiome and cancer progression (172). The study by Canale et al. can provide a way forward (171). Increase intra-tumoral L-arginine levels is a key determinant of an efficient anti-tumor T cell response (173). Canale et al. reported that they used the synthetic biology approach to develop an engineered probiotic *Escherichia coli Nissle 1917* strain that colonizes tumors and continuously converts ammonia to L-arginine. There is a growing recognition that using live engineered bacteria for metabolic modulation is a new strategy for cancer immunotherapy (174). Moreover, He et al. reported that mice infused back with butyric acid or supplemented with intestinal flora were also able to promote CD8^+^ T-cell infiltration and function in tumors and rescue the efficacy of the chemotherapeutic agent oxaliplatin (175). Moreover, a recent study showed that Pien Tze Huang suppresses colorectal tumorigenesis through restoring gut microbiota and metabolites (176). Traditional medicine regulated the crosstalk between microbiota and metabolites, which provided novel ideas for clinical treatment of cancer. Some scholars attempted to use dietary improvement (177, 178), utilizing phages to eliminate certain microorganisms which secret metabolites (179), fecal microbiota transplantation (180), and so on for cancer treatment by modulating the crosstalk between microbiota and metabolic reprogramming (**Figure 5**). Understanding the complex relationship between microbes and metabolic reprogramming could provide valuable insights into potential cancer treatment options.

## Concluding remarks

Metabolic reprogramming plays a critical role in the maintenance of increased nutrient demands for cancer cells. Metabolic reprogramming is a hallmark of cancer and often observed in human malignancy. Therefore, it is speculated that certain metabolism can play a regulatory role, as it can promote tumor growth or inhibit tumor cells by providing metabolic energy or activating certain intracellular signaling pathways. The complex community of microorganisms found in the human body has the potential to regulate tumor progression and the treatment response of multiple types of cancer. More importantly, bacteria have been globally proven to be involved in the progression of cancer via metabolic regulation, suggesting a complex interaction between metabolic reprogramming and microbiota. Despite significant progress, studies on microbiota and metabolic reprogramming face several critical limitations. Firstly, while high-throughput sequencing (16S rRNA or metagenomics) reveals associations between microbial composition and metabolic phenotypes, it cannot prove direct mechanistic links. Mice models often fail to fully recapitulate human host-microbe interactions due to interspecies differences and individual variability. Secondly, the factors like age, diet, geography, and genetics lead to substantial baseline variations, making it difficult to define a “healthy” microbiome. Additionally, microbial communities are highly dynamic and sensitive to short-term perturbations (such as antibiotic use or dietary changes), requiring complex longitudinal study designs. Thirdly, a single bacterial species may influence the host through multiple metabolites, and host-microbe co-metabolism is challenging to replicate *in vitro*. Additionally, the current technology cannot distinguish live/dead bacteria and lacks sensitivity for low-abundance taxa. Finally, clinical translation faces barriers, with current interventions showing inconsistent efficacy and safety risks that need to be carefully evaluated. Thus, prolonged efforts will be required to solve the problems to create the best therapeutic protocols that can bring a new hope for patients with cancer.

This review explains how microbiota regulate the development of malignancy by the metabolism and summarizes the influence of various bacteria-related metabolic reprogramming mechanisms on the biological behavior of cancer. Considering that both metabolic reprogramming and microbiota play a key role in tumor progression and that crosstalk between them has been discovered recently, targeting metabolism and/or microbiota with drugs may be beneficial for controlling tumor development. We believe that the unique metabolic pathways related to the certain microbiota; identifying the crosstalk between them and exploring their mechanisms will advance the field of cancer research.
